# Supplementary material for: Modelling Skylarks (Alauda arvensis) to Predict Impacts of Changes in Land Management and Policy: Development and Testing of an Agent-Based Model
Source: PLoS One. 2013 Jun 6;8(6):e65803. doi: 10.1371/journal.pone.0065803 (PMC3675089; doi:10.1371/journal.pone.0065803)
Supplement: Supporting Information S4 — The skylark ODdox as a zipped archive. (ZIP) [file pone.0065803.s004.zip › Skylark_ODdox/class_t_a_l_ma_s_s_object.html]

ALMaSS Skylark ODdox: TALMaSSObject Class Reference


ALMaSS Skylark ODdox
 2.0


- Main Page
- Related Pages
- Classes
- Files

- Class List
- Class Index
- Class Hierarchy
- Class Members

Public Member Functions |
Public Attributes

TALMaSSObject Class Reference

The base class of all ALMaSS objects requiring Step code.
More...

`#include <populationmanager.h>`

List of all members.

|  |  |
| --- | --- |
| Public Member Functions | |
| virtual void | BeginStep (void) |
| virtual void | EndStep (void) |
| void | OnArrayBoundsError () |
| virtual void | Step (void) |
|  | TALMaSSObject () |
| virtual | ~TALMaSSObject () |

|  |  |
| --- | --- |
| Public Attributes | |
| int | CurrentStateNo |
| bool | StepDone |

---

## Detailed Description

The base class of all ALMaSS objects requiring Step code.

---

## Constructor & Destructor Documentation

|  |  |  |  |  |
| --- | --- | --- | --- | --- |
| TALMaSSObject::TALMaSSObject | ( |  | ) |  |

TALMaSSObject Constructor

{

#ifdef \_\_CJTDebug\_5

AmAlive = 0xDEADC0DE;

#endif

StepDone = false;

CurrentStateNo = 0;

}

|  |  |  |  |  |
| --- | --- | --- | --- | --- |
| TALMaSSObject::~TALMaSSObject | ( |  | ) |  |

virtual

TALMaSSObject Destructor

{

#ifdef \_\_CJTDebug\_5

AmAlive = 0;

#endif

}

---

## Member Function Documentation

|  |  |  |  |  |  |
| --- | --- | --- | --- | --- | --- |
| virtual void TALMaSSObject::BeginStep | ( | void |  | ) |  |

inlinevirtual

Reimplemented in Skylark\_Male, Skylark\_Female, Skylark\_PreFledgeling, Skylark\_Nestling, Skylark\_Clutch, and TAnimal.

{

}

|  |  |  |  |  |  |
| --- | --- | --- | --- | --- | --- |
| virtual void TALMaSSObject::EndStep | ( | void |  | ) |  |

inlinevirtual

Reimplemented in Skylark\_Male, Skylark\_Female, Skylark\_PreFledgeling, Skylark\_Nestling, Skylark\_Clutch, and TAnimal.

{

}

|  |  |  |  |  |
| --- | --- | --- | --- | --- |
| void TALMaSSObject::OnArrayBoundsError | ( |  | ) |  |

Referenced by Skylark\_Male::GetFood(), Skylark\_Male::OnAddNestling(), Skylark\_Male::OnNestlingDeath(), Skylark\_Male::OnPreFledgelingDeath(), and Skylark\_Male::OnPreFledgelingMature().

{

exit( 1 );

}

|  |  |  |  |  |  |
| --- | --- | --- | --- | --- | --- |
| virtual void TALMaSSObject::Step | ( | void |  | ) |  |

inlinevirtual

Reimplemented in Skylark\_Male, Skylark\_Female, Skylark\_PreFledgeling, Skylark\_Nestling, Skylark\_Clutch, and TAnimal.

{

}

---

## Member Data Documentation

int TALMaSSObject::CurrentStateNo

Referenced by Skylark\_Nestling::BeginStep(), Skylark\_Clutch::EndStep(), Skylark\_Nestling::EndStep(), Skylark\_PreFledgeling::EndStep(), TAnimal::KillThis(), Skylark\_Nestling::OnDeserted(), Skylark\_Clutch::OnMumGone(), Skylark\_Nestling::OnYouHaveBeenEaten(), CompareStateR::operator()(), Skylark\_Base::Skylark\_Base(), Skylark\_Clutch::st\_Dying(), Skylark\_Nestling::st\_Dying(), Skylark\_PreFledgeling::st\_Dying(), Skylark\_Female::st\_Dying(), and Skylark\_Male::st\_Dying().

bool TALMaSSObject::StepDone

Referenced by Skylark\_Female::BeginStep(), Skylark\_Male::BeginStep(), Skylark\_Clutch::EndStep(), Skylark\_Nestling::EndStep(), Skylark\_PreFledgeling::EndStep(), TAnimal::KillThis(), Skylark\_Clutch::Step(), Skylark\_Nestling::Step(), Skylark\_PreFledgeling::Step(), Skylark\_Female::Step(), and Skylark\_Male::Step().

---

The documentation for this class was generated from the following files:

- populationmanager.h
- PopulationManager.cpp


- TALMaSSObject
- Generated on Thu Jan 10 2013 13:15:38 for ALMaSS Skylark ODdox by
   1.8.1.1
